# The trigonal polymorph of strontium tetra­borate, β-SrB_4_O_7_

**DOI:** 10.1107/S1600536810019069

**Published:** 2010-05-29

**Authors:** Alexander D. Vasiliev, Alexander V. Cherepakhin, Alexander I. Zaitsev

**Affiliations:** a Institute of Physics SB RAS, Krasnoyarsk 660036, Russian Federation and Siberian Federal University, Svobodnyi st. 79, Krasnoyarsk 660041, Russian Federation

## Abstract

The asymmetric unit of the title compound, β-SrB_4_O_7_, contains five Sr atoms (three located on a threefold rotation axis), twelve B and 21 O atoms. The structure is made up from BO_3_ triangles and BO_4_ tetra­hedra in a 1:1 ratio. Pairs of BO_3_ triangles are linked to BO_4_ tetra­hedra *via* common corners, forming chains. These chains are further linked to adjacent chains through corner-sharing, leading to a three-dimensional framework with channels running parallel to [001]. The Sr^2+^ ions reside in the channels and exhibit strongly distorted polyhedra The density of the β-polymorph is considerably lower than that of α-SrB_4_O_7_, which is constructed solely from BO_4_ tetra­hedra.

## Related literature

For the ortho­rhom­bic α-polymorph, see: Block *et al.* (1964 ▶). For the physical properties of this phase, see: Oseledchik *et al.* (1995 ▶); Petrov *et al.* (2004 ▶); Zaitsev *et al.* (2006 ▶); Verwey *et al.* (1992 ▶); Machida *et al.* (1979 ▶); Pei *et al.* (2000 ▶). For other crystalline phases in the system SrO—B_2_O_3_ listed in the ICSD (2009 ▶), see: Ross & Angel (1991 ▶); Lin *et al.* (1999 ▶); Wei *et al.* (2001 ▶); Tang *et al.* (2008 ▶); Lapshin *et al.* (2007 ▶); Kim *et al.* (1996 ▶). For glass-phases in this system, see: Imaoka (1959 ▶); Polyakova & Litovchik (2008 ▶). For a review of B—O bond lengths in BO_3_ and BO_4_ units, see: Zobetz (1982 ▶, 1990 ▶).

## Experimental

### 

#### Crystal data

SrB_4_O_7_
*M* *_r_* = 242.86
Trigonal, 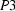

*a* = 17.145 (1) Å
*c* = 4.2527 (5) Å
*V* = 1082.61 (16) Å^3^
*Z* = 9
Mo *K*α radiation
μ = 11.19 mm^−1^
*T* = 296 K
0.40 × 0.25 × 0.18 mm

#### Data collection

Bruker SMART CCD area-detector diffractometer
Absorption correction: multi-scan (*SADABS*; Sheldrick, 2004 ▶) *T*_min_ = 0.095, *T*_max_ = 0.242
10350 measured reflections
3709 independent reflections
3202 reflections with *I* > 2σ(*I*)
*R*_int_ = 0.054

#### Refinement

*R*[*F* ^2^ > 2σ(*F* ^2^)] = 0.030
*wR*(*F* ^2^) = 0.064
*S* = 0.85
3709 reflections
265 parameters
Δρ_max_ = 1.02 e Å^−3^
Δρ_min_ = −0.49 e Å^−3^
Absolute structure: Flack (1983 ▶), 1836 Friedel pairs
Flack parameter: −0.030 (7)

### 

Data collection: *SMART* (Bruker, 2001 ▶); cell refinement: *SAINT*-*Plus* (Bruker, 2001 ▶); data reduction: *SAINT*-*Plus*; program(s) used to solve structure: *SHELXTL* (Sheldrick, 2008 ▶); program(s) used to refine structure: *SHELXTL*; molecular graphics: *SHELXTL*; software used to prepare material for publication: *SHELXTL*.

## Supplementary Material

Crystal structure: contains datablocks global, I. DOI: 10.1107/S1600536810019069/wm2345sup1.cif

Structure factors: contains datablocks I. DOI: 10.1107/S1600536810019069/wm2345Isup2.hkl

Additional supplementary materials:  crystallographic information; 3D view; checkCIF report

## Figures and Tables

**Fig. 1.**
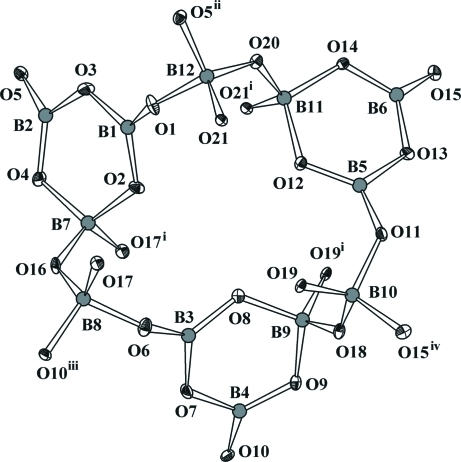
View of the basic structural motif in the title compound with the atom numbering scheme. Displacement ellipsoids are drawn at the 50% probability level. Symmetry codes: (i) x, y, z-1; (ii) 1-y, 1+x-y, 1+z; (iii) -y, x-y, 1+z; (iv) 1-y, x-y, 1+z.

**Fig. 2.**
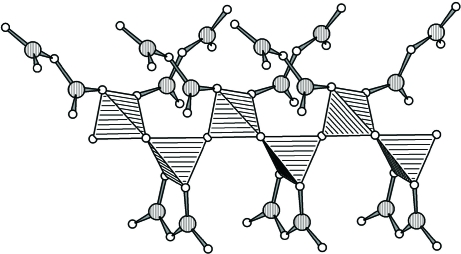
Columns of BO4 tetrahedra with shared vertices and attached BO3 triangles extending along the c-direction.

**Fig. 3.**
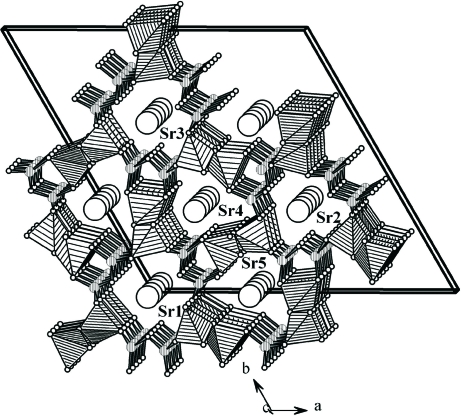
View down [001] of the framework structure of the title compound showing the formation of channels that are filled with Sr2+ ions.

## supplementary crystallographic information

### Comment

The orthorhombic phase of strontium tetraborate, α-SrB_4_O_7_ (I), is known for a long time (Block *et al.*, 1964). This compound has attracted attention owing to its interesting physical properties, namely an unprecedented fundamental optical-absorption edge among oxide compounds (~130 nm), high non-linear optical coefficients (Oseledchik *et al.*, 1995; Petrov *et al.*, 2004; Zaitsev *et al.*, 2006), good luminescent characteristics and an ability to stabilize rare-earth elements in divalent state (Verwey *et al.*, 1992; Pei *et al.*, 2000; Machida *et al.*, 1979).

SrB_4_O_7_ falls in a glass-forming range within the SrO—B_2_O_3_ system and can simply be obtained as a glass (Imaoka, 1959). The process of glass re-crystallization occurs through complex mechanisms with probabilistic formation of other crystalline phases, specifically of metastable phases. Such a phase was in fact observed and designated as β-SrB_4_O_7_ (Polyakova & Litovchik, 2008). However, X-ray powder diffraction data of this phase and of two other new compounds described by these authors were not analysed because of impure samples.

The FIZ/NIST Inorganic Crystal Structure Database (release 2009; ICSD, 2009) reveals six phases in the SrO—B_2_O_3_ system besides (I): strontium diborate, (IIa), SrB_2_O_4_ (Kim *et al.*, 1996), its high-pressure form (Ross & Angel, 1991), (IIb), distrontium diborate, Sr_2_B_2_O_5_, (III), (Lin *et al.*, 1999), tristrontium tetraborate, Sr_3_B_2_O_6_, (IV), (Wei *et al.*, 2001), distrontium hexadecaborate, Sr_2_B_16_O_26_, (V), (Tang *et al.*, 2008) and tetrastrontium tetradecaborate, Sr_4_B_14_O_25_, (VI), (Lapshin *et al.*, 2007). Only two of these phases crystallize in non-centrosymmetric space groups, *viz*. (I, *Pmn*2_1_ and VI, *Cmc*2_1_). The main feature of all these structures are BO*_x_* units (*x* = 3,4). Isolated (IV) or flat pairs (III) of BO_3_ triangles, a framework of BO_4_ tetrahedra with shared vertices (I, II) and a framework of triangles and tetrahedra with shared vertices (V, VI) are found in these structures.

In the process of glass re-crystallization of a strontium tetraborate composition at 973–983 K during one day, we obtained β-SrB_4_O_7_ crystals with dimensions of ~200–400 µm. The crystals were located on the glass surface and were optically homogeneous (i.e. crystals showed homogeneous extinction when observed under a polarizing microscope). The crystals possess strong anisotropy when abrased, and crystals with an elongated ellipsoidal shape were obtained in such a way. As it turned out, the crystals are most firm along the *c*-direction.

The crystal structure of (I) is built up from a three-dimensional framework of connected boron-oxygen tetrahedra. The asymmetric unit of the title structure contains five Sr (three on special positions), twelve B and 21 O atoms. Alternatively, the structural formula of the title compound can thus be written as Sr_3_B_12_O_21_. It consists of BO_3_ triangles and BO_4_ tetrahedra in an 1:1 ratio (3:1 for structure V and 3:4 for structure VI). They form a three-dimensional framework constructed *via* common vertices. The BO_3_ triangles are linked to one another so that two of their vertices and the bridging O atom are located on a straight line (see O1, O3, O5; O6, O7, O10 and O11, O13, O15 in Fig.1). The plane of one triangle in such a pair is tilted relatively to the other one about the line with an angle of ~20°. The remaining two vertices are common with the same tetrahedron (e.g. see O2 and O4 in Fig.1). The BO_4_ tetrahedra are connected to one another *via* common vertices and form chains along the *c*-direction (Fig. 2). These chains are connected with pairs of BO_3_ triangles, leading to the formation of channels in the structure. The channels are filled with strontium ions (Fig. 3). The coordination polyhedra around the strontium ions are non-regular and defined by six O atoms in the range 2.479 (3)–2.786 (3) Å when a distance < 2.8 Å is considered as relevant.

All vertices in the anionic framework are shared so that every oxygen atom is connected to two boron atoms. The B—O distances fall into the interval 1.323 (6)–1.420 (6)Å (average is 1.367 (6) Å) for BO_3_ triangles and into the interval 1.425 (6)–1.538 (6) Å (average is 1.474 (6) Å) for BO_4_ tetrahedra. These values compare well with the mean bond lengths calculated for various borate structures (Zobetz, 1982, 1990).

In comparison with α-SrB_4_O_7_ which is constructued solely from BO_4_ tetrahedra, the density of the β-polymorph is considerably lower.

### Experimental

Crystals were extracted out of glass by careful dissolving of the latter in a 2% HNO_3_ solution. The initial glass has been made from a mixture of SrCO_3_ (99.8%) and H_3_BO_3_ (99.98%) in a 1:4 ratio. The mixture was heated up to 353—363 K with addition of a small amount of water and careful mixing until CO_2_ gas evolution had stopped. Then the temperature was increased slowly up to 573 K to yield a anhydrous phase. The derived mixture was then placed into a glass-carbon crucible and kept in a molten state at 1323 K during 6 h in a nitrogen atmosphere. The flux was cooled in air down to 773 K and the glass was finally annealed at 723 K during a day to remove strain.

### Crystal data

**Table:**

SrB_4_O_7_	*D*_x_ = 3.353 (1) Mg m^−^^3^
*M**_r_* = 242.86	Mo *K*α radiation, λ = 0.71073 Å
Trigonal, *P*3	Cell parameters from 2820 reflections
Hall symbol: P 3	θ = 2.4–29.3°
*a* = 17.145 (1) Å	µ = 11.19 mm^−^^1^
*c* = 4.2527 (5) Å	*T* = 296 K
*V* = 1082.61 (16) Å^3^	Ellipsoidal, colorless
*Z* = 9	0.40 × 0.25 × 0.18 mm
*F*(000) = 1026	

### Data collection

**Table:**

Bruker SMART CCD area-detector diffractometer	3709 independent reflections
Radiation source: fine-focus sealed tube	3202 reflections with *I* > 2σ(*I*)
graphite	*R*_int_ = 0.054
φ and ω scans	θ_max_ = 28.7°, θ_min_ = 2.4°
Absorption correction: multi-scan (*SADABS*; Sheldrick, 2004)	*h* = −22→23
*T*_min_ = 0.095, *T*_max_ = 0.242	*k* = −23→23
10350 measured reflections	*l* = −5→5

### Refinement

**Table:**

Refinement on *F*^2^	Secondary atom site location: difference Fourier map
Least-squares matrix: full	*w* = 1/[σ^2^(*F*_o_^2^) + (0.*P*)^2^] where *P* = (*F*_o_^2^ + 2*F*_c_^2^)/3
*R*[*F*^2^ > 2σ(*F*^2^)] = 0.030	(Δ/σ)_max_ < 0.001
*wR*(*F*^2^) = 0.064	Δρ_max_ = 1.02 e Å^−^^3^
*S* = 0.85	Δρ_min_ = −0.49 e Å^−^^3^
3709 reflections	Extinction correction: *SHELXTL* (Sheldrick, 2008), Fc^*^=kFc[1+0.001xFc^2^λ^3^/sin(2θ)]^-1/4^
265 parameters	Extinction coefficient: 0.0794 (13)
0 restraints	Absolute structure: Flack (1983), 1836 Friedel pairs
Primary atom site location: structure-invariant direct methods	Flack parameter: −0.030 (7)

### Special details

**Table:**

Geometry. All esds (except the esd in the dihedral angle between two l.s. planes) are estimated using the full covariance matrix. The cell esds are taken into account individually in the estimation of esds in distances, angles and torsion angles; correlations between esds in cell parameters are only used when they are defined by crystal symmetry. An approximate (isotropic) treatment of cell esds is used for estimating esds involving l.s. planes.
Refinement. Refinement of *F*^2^ against ALL reflections. The weighted *R*-factor wR and goodness of fit *S* are based on *F*^2^, conventional *R*-factors *R* are based on *F*, with *F* set to zero for negative *F*^2^. The threshold expression of *F*^2^ > σ(*F*^2^) is used only for calculating *R*-factors(gt) etc. and is not relevant to the choice of reflections for refinement. *R*-factors based on *F*^2^ are statistically about twice as large as those based on *F*, and *R*- factors based on ALL data will be even larger.

### Fractional atomic coordinates and isotropic or equivalent isotropic displacement parameters (Å^2^)

**Table:**

	*x*	*y*	*z*	*U*_iso_*/*U*_eq_	
Sr1	0.0000	0.0000	0.5000	0.0106 (2)	
Sr2	0.6667	0.3333	0.7047 (2)	0.0097 (2)	
Sr3	0.3333	0.6667	0.6084 (5)	0.01172 (15)	
Sr4	0.32408 (4)	0.33158 (4)	0.7001 (4)	0.00732 (14)	
Sr5	−0.00569 (3)	0.33541 (4)	0.5183 (4)	0.00743 (15)	
O1	0.3441 (2)	0.5031 (2)	0.5634 (9)	0.0115 (7)	
O2	0.2472 (2)	0.3798 (2)	0.2476 (8)	0.0094 (7)	
O3	0.2591 (2)	0.5240 (2)	0.1833 (8)	0.0108 (7)	
O4	0.1239 (2)	0.3939 (2)	0.0012 (9)	0.0109 (7)	
O5	0.1760 (2)	0.5441 (2)	−0.1939 (8)	0.0090 (7)	
O6	0.1571 (2)	0.1803 (2)	0.4810 (9)	0.0119 (8)	
O7	0.2841 (2)	0.2150 (2)	0.1685 (8)	0.0075 (6)	
O8	0.1428 (2)	0.0790 (2)	0.0952 (8)	0.0109 (7)	
O9	0.2767 (2)	0.0826 (2)	−0.0917 (8)	0.0083 (7)	
O10	0.1278 (2)	−0.0221 (2)	−0.2863 (8)	0.0095 (7)	
O11	0.5201 (2)	0.3007 (2)	0.4426 (8)	0.0082 (7)	
O12	0.4709 (2)	0.4061 (2)	0.3108 (8)	0.0077 (7)	
O13	0.6000 (2)	0.4102 (2)	0.0726 (8)	0.0105 (7)	
O14	0.5899 (2)	0.5436 (2)	0.0455 (8)	0.0065 (6)	
O15	0.6814 (2)	0.5195 (2)	−0.3106 (8)	0.0082 (7)	
O16	0.0899 (2)	0.2724 (2)	0.3487 (8)	0.0084 (7)	
O17	0.1632 (2)	0.2814 (2)	0.8498 (8)	0.0085 (7)	
O18	0.3864 (2)	0.1604 (2)	0.2995 (8)	0.0082 (7)	
O19	0.3945 (2)	0.2362 (2)	0.7992 (8)	0.0062 (6)	
O20	0.5007 (2)	0.5578 (2)	0.4336 (8)	0.0073 (7)	
O21	0.4320 (2)	0.4828 (2)	0.9349 (8)	0.0067 (7)	
B1	0.2862 (4)	0.4681 (4)	0.3246 (14)	0.0079 (10)*	
B2	0.1811 (3)	0.4849 (3)	−0.0018 (13)	0.0059 (10)*	
B3	0.1948 (3)	0.1620 (3)	0.2422 (13)	0.0075 (10)*	
B4	0.1853 (4)	0.0444 (3)	−0.0934 (13)	0.0071 (10)*	
B5	0.5250 (3)	0.3704 (3)	0.2794 (12)	0.0052 (9)*	
B6	0.6277 (3)	0.4939 (3)	−0.0547 (13)	0.0063 (10)*	
B7	0.1537 (4)	0.3283 (3)	0.1104 (13)	0.0048 (10)*	
B8	0.1082 (3)	0.2266 (3)	0.5981 (12)	0.0052 (10)*	
B9	0.3394 (4)	0.1748 (4)	0.0454 (12)	0.0064 (11)*	
B10	0.4409 (3)	0.2160 (3)	0.5607 (12)	0.0063 (10)*	
B11	0.4960 (4)	0.4991 (4)	0.1768 (13)	0.0076 (12)*	
B12	0.4379 (3)	0.5431 (3)	0.6829 (12)	0.0061 (10)*	

### Atomic displacement parameters (Å^2^)

**Table:**

	*U*^11^	*U*^22^	*U*^33^	*U*^12^	*U*^13^	*U*^23^
Sr1	0.0098 (3)	0.0098 (3)	0.0121 (5)	0.00490 (15)	0.000	0.000
Sr2	0.0093 (3)	0.0093 (3)	0.0106 (5)	0.00466 (16)	0.000	0.000
Sr3	0.0100 (2)	0.0100 (2)	0.0151 (4)	0.00502 (10)	0.000	0.000
Sr4	0.0072 (3)	0.0066 (3)	0.0083 (3)	0.0036 (2)	0.0001 (3)	0.0014 (2)
Sr5	0.0072 (3)	0.0076 (3)	0.0082 (3)	0.0043 (2)	0.0007 (2)	0.0015 (2)
O1	0.0055 (16)	0.0191 (19)	0.0082 (17)	0.0048 (15)	−0.0004 (14)	−0.0017 (14)
O2	0.0069 (16)	0.0074 (16)	0.0109 (17)	0.0014 (13)	−0.0039 (13)	0.0001 (13)
O3	0.0099 (16)	0.0065 (16)	0.0141 (18)	0.0027 (14)	−0.0075 (14)	−0.0033 (14)
O4	0.0090 (16)	0.0103 (17)	0.0153 (19)	0.0062 (14)	−0.0051 (14)	−0.0006 (14)
O5	0.0074 (16)	0.0100 (17)	0.0091 (18)	0.0039 (14)	−0.0009 (13)	0.0018 (13)
O6	0.0139 (19)	0.0164 (18)	0.0101 (18)	0.0110 (16)	0.0039 (14)	0.0042 (15)
O7	0.0080 (16)	0.0090 (16)	0.0058 (16)	0.0046 (14)	−0.0001 (13)	−0.0004 (13)
O8	0.0065 (16)	0.0099 (16)	0.0148 (19)	0.0029 (14)	0.0034 (14)	−0.0030 (14)
O9	0.0076 (16)	0.0109 (16)	0.0081 (16)	0.0059 (14)	0.0012 (13)	−0.0006 (13)
O10	0.0087 (17)	0.0060 (16)	0.0148 (19)	0.0044 (14)	−0.0046 (14)	−0.0022 (14)
O11	0.0061 (16)	0.0095 (16)	0.0093 (17)	0.0042 (14)	0.0016 (13)	0.0018 (13)
O12	0.0090 (16)	0.0090 (16)	0.0069 (16)	0.0057 (14)	0.0031 (13)	0.0025 (13)
O13	0.0104 (17)	0.0078 (16)	0.0125 (18)	0.0039 (14)	0.0087 (13)	0.0050 (13)
O14	0.0077 (15)	0.0062 (15)	0.0060 (16)	0.0039 (13)	0.0001 (13)	−0.0009 (12)
O15	0.0086 (18)	0.0114 (18)	0.0048 (17)	0.0053 (15)	0.0040 (14)	0.0046 (14)
O16	0.0069 (16)	0.0130 (17)	0.0075 (16)	0.0066 (14)	0.0036 (13)	0.0026 (13)
O17	0.0082 (16)	0.0092 (16)	0.0077 (17)	0.0041 (14)	−0.0004 (13)	−0.0023 (13)
O18	0.0081 (16)	0.0112 (16)	0.0079 (17)	0.0068 (14)	−0.0016 (13)	0.0003 (13)
O19	0.0089 (15)	0.0083 (16)	0.0043 (16)	0.0065 (13)	0.0021 (12)	0.0017 (12)
O20	0.0099 (16)	0.0099 (16)	0.0038 (15)	0.0061 (14)	0.0024 (13)	0.0000 (12)
O21	0.0057 (15)	0.0066 (15)	0.0076 (17)	0.0029 (13)	−0.0009 (12)	0.0029 (13)

### Geometric parameters (Å, °)

**Table:**

Sr1—O10^i^	2.569 (3)	Sr5—B11^xii^	3.171 (6)
Sr1—O10^ii^	2.569 (3)	O1—B1	1.335 (6)
Sr1—O10^iii^	2.569 (3)	O1—B12	1.486 (6)
Sr1—O8^iv^	2.734 (3)	O2—B1	1.354 (6)
Sr1—O8^v^	2.734 (3)	O2—B7	1.509 (6)
Sr1—O8	2.734 (3)	O2—Sr4^xiv^	2.988 (3)
Sr1—O6	2.913 (4)	O3—B1	1.392 (6)
Sr1—O6^v^	2.913 (4)	O3—B2	1.401 (6)
Sr1—O6^iv^	2.913 (4)	O3—Sr3^xiv^	3.236 (3)
Sr1—B3	3.285 (5)	O4—B2	1.366 (6)
Sr1—B3^iv^	3.285 (5)	O4—B7	1.521 (6)
Sr1—B3^v^	3.285 (5)	O4—Sr5^xiv^	2.816 (3)
Sr2—O11^vi^	2.542 (3)	O5—B2	1.340 (6)
Sr2—O11^vii^	2.542 (3)	O5—B12^xv^	1.489 (6)
Sr2—O11	2.542 (3)	O5—Sr3^xiv^	2.594 (3)
Sr2—O13^viii^	2.644 (3)	O6—B3	1.323 (6)
Sr2—O13^ix^	2.644 (3)	O6—B8	1.499 (6)
Sr2—O13^ii^	2.644 (3)	O7—B3	1.370 (6)
Sr2—O15^viii^	3.074 (3)	O7—B9	1.517 (6)
Sr2—O15^ix^	3.074 (3)	O7—Sr4^xiv^	2.657 (3)
Sr2—O15^ii^	3.074 (3)	O8—B3	1.394 (6)
Sr2—B6^viii^	3.303 (5)	O8—B4	1.399 (6)
Sr2—B6^ix^	3.303 (5)	O8—Sr1^xiv^	3.304 (3)
Sr2—B6^ii^	3.303 (5)	O9—B4	1.364 (6)
Sr3—O5^x^	2.594 (3)	O9—B9	1.516 (6)
Sr3—O5^ii^	2.594 (3)	O9—Sr5^xvi^	2.676 (3)
Sr3—O5^xi^	2.594 (3)	O10—B4	1.349 (6)
Sr3—O3^xii^	2.786 (3)	O10—B8^xvi^	1.487 (5)
Sr3—O3	2.786 (3)	O10—Sr1^xiv^	2.569 (3)
Sr3—O3^xiii^	2.786 (3)	O11—B5	1.348 (6)
Sr3—O1^xii^	2.908 (3)	O11—B10	1.494 (6)
Sr3—O1^xiii^	2.908 (3)	O12—B5	1.350 (6)
Sr3—O1	2.908 (3)	O12—B11	1.537 (6)
Sr3—O3^x^	3.236 (3)	O13—B6	1.377 (6)
Sr3—O3^xi^	3.236 (4)	O13—B5	1.419 (6)
Sr3—O3^ii^	3.236 (4)	O13—Sr2^xiv^	2.644 (3)
Sr4—O19	2.507 (3)	O14—B6	1.371 (6)
Sr4—O21	2.519 (3)	O14—B11	1.503 (6)
Sr4—O17	2.526 (3)	O14—Sr5^xiii^	2.657 (3)
Sr4—O7^ii^	2.657 (3)	O14—Sr5^xvii^	2.836 (3)
Sr4—O2	2.685 (3)	O15—B6	1.349 (6)
Sr4—O12	2.737 (3)	O15—B10^xviii^	1.501 (6)
Sr4—O1	2.845 (4)	O15—Sr5^xvii^	2.649 (3)
Sr4—O7	2.864 (3)	O15—Sr2^xiv^	3.074 (3)
Sr4—O6	2.893 (4)	O16—B8	1.445 (6)
Sr4—O2^ii^	2.988 (3)	O16—B7	1.447 (6)
Sr4—B12	3.145 (5)	O17—B8	1.425 (6)
Sr4—B1	3.158 (5)	O17—B7^ii^	1.426 (6)
Sr5—O16	2.479 (3)	O18—B9	1.441 (6)
Sr5—O20^xii^	2.482 (3)	O18—B10	1.458 (6)
Sr5—O18^v^	2.539 (3)	O18—Sr5^iv^	2.539 (3)
Sr5—O15^x^	2.649 (3)	O19—B10	1.434 (6)
Sr5—O14^xii^	2.657 (3)	O19—B9^ii^	1.450 (5)
Sr5—O9^i^	2.676 (3)	O20—B12	1.440 (6)
Sr5—O4^ii^	2.816 (3)	O20—B11	1.460 (6)
Sr5—O14^x^	2.836 (3)	O20—Sr5^xiii^	2.482 (3)
Sr5—O4	2.924 (4)	O21—B11^ii^	1.425 (6)
Sr5—B10^v^	3.129 (5)	O21—B12	1.458 (6)
Sr5—B6^x^	3.149 (5)		
			
O10^i^—Sr1—O10^ii^	108.20 (8)	O7—Sr4—O2^ii^	145.41 (8)
O10^i^—Sr1—O10^iii^	108.20 (8)	O6—Sr4—O2^ii^	97.10 (9)
O10^ii^—Sr1—O10^iii^	108.20 (8)	O16—Sr5—O20^xii^	116.34 (10)
O10^i^—Sr1—O8^iv^	155.68 (11)	O16—Sr5—O18^v^	104.21 (10)
O10^ii^—Sr1—O8^iv^	94.57 (10)	O20^xii^—Sr5—O18^v^	119.04 (10)
O10^iii^—Sr1—O8^iv^	71.17 (10)	O16—Sr5—O15^x^	152.34 (10)
O10^i^—Sr1—O8^v^	71.17 (10)	O20^xii^—Sr5—O15^x^	90.34 (10)
O10^ii^—Sr1—O8^v^	155.68 (11)	O18^v^—Sr5—O15^x^	52.72 (10)
O10^iii^—Sr1—O8^v^	94.57 (10)	O16—Sr5—O14^xii^	112.32 (11)
O8^iv^—Sr1—O8^v^	84.57 (11)	O20^xii^—Sr5—O14^xii^	54.17 (10)
O10^i^—Sr1—O8	94.57 (10)	O18^v^—Sr5—O14^xii^	69.52 (10)
O10^ii^—Sr1—O8	71.17 (10)	O15^x^—Sr5—O14^xii^	76.45 (10)
O10^iii^—Sr1—O8	155.68 (11)	O16—Sr5—O9^i^	85.40 (10)
O8^iv^—Sr1—O8	84.57 (11)	O20^xii^—Sr5—O9^i^	148.56 (12)
O8^v^—Sr1—O8	84.57 (11)	O18^v^—Sr5—O9^i^	72.45 (10)
O10^i^—Sr1—O6	49.69 (9)	O15^x^—Sr5—O9^i^	73.60 (10)
O10^ii^—Sr1—O6	76.23 (9)	O14^xii^—Sr5—O9^i^	140.89 (9)
O10^iii^—Sr1—O6	156.14 (11)	O16—Sr5—O4^ii^	77.96 (10)
O8^iv^—Sr1—O6	132.54 (10)	O20^xii^—Sr5—O4^ii^	83.34 (10)
O8^v^—Sr1—O6	86.46 (9)	O18^v^—Sr5—O4^ii^	151.29 (11)
O8—Sr1—O6	48.18 (9)	O15^x^—Sr5—O4^ii^	114.40 (11)
O10^i^—Sr1—O6^v^	76.23 (9)	O14^xii^—Sr5—O4^ii^	136.93 (9)
O10^ii^—Sr1—O6^v^	156.14 (11)	O9^i^—Sr5—O4^ii^	79.29 (10)
O10^iii^—Sr1—O6^v^	49.69 (9)	O16—Sr5—O14^x^	142.36 (10)
O8^iv^—Sr1—O6^v^	86.46 (9)	O20^xii^—Sr5—O14^x^	70.68 (10)
O8^v^—Sr1—O6^v^	48.18 (9)	O18^v^—Sr5—O14^x^	102.82 (9)
O8—Sr1—O6^v^	132.54 (10)	O15^x^—Sr5—O14^x^	50.85 (9)
O6—Sr1—O6^v^	119.923 (7)	O14^xii^—Sr5—O14^x^	101.42 (10)
O10^i^—Sr1—O6^iv^	156.14 (11)	O9^i^—Sr5—O14^x^	78.32 (9)
O10^ii^—Sr1—O6^iv^	49.69 (9)	O4^ii^—Sr5—O14^x^	65.84 (10)
O10^iii^—Sr1—O6^iv^	76.23 (9)	O16—Sr5—O4	51.39 (10)
O8^iv^—Sr1—O6^iv^	48.18 (9)	O20^xii^—Sr5—O4	71.17 (10)
O8^v^—Sr1—O6^iv^	132.54 (10)	O18^v^—Sr5—O4	108.16 (10)
O8—Sr1—O6^iv^	86.46 (9)	O15^x^—Sr5—O4	142.90 (10)
O6—Sr1—O6^iv^	119.923 (8)	O14^xii^—Sr5—O4	66.60 (9)
O6^v^—Sr1—O6^iv^	119.923 (8)	O9^i^—Sr5—O4	136.26 (9)
O11^vi^—Sr2—O11^vii^	102.21 (9)	O4^ii^—Sr5—O4	95.60 (10)
O11^vi^—Sr2—O11	102.21 (9)	O14^x^—Sr5—O4	139.09 (9)
O11^vii^—Sr2—O11	102.21 (9)	B1—O1—B12	149.0 (4)
O11^vi^—Sr2—O13^viii^	94.41 (10)	B1—O1—Sr4	90.7 (3)
O11^vii^—Sr2—O13^viii^	75.13 (10)	B12—O1—Sr4	87.2 (2)
O11—Sr2—O13^viii^	163.33 (10)	B1—O1—Sr3	95.2 (3)
O11^vi^—Sr2—O13^ix^	75.13 (10)	B12—O1—Sr3	96.2 (3)
O11^vii^—Sr2—O13^ix^	163.33 (10)	Sr4—O1—Sr3	161.90 (14)
O11—Sr2—O13^ix^	94.41 (10)	B1—O2—B7	122.2 (4)
O13^viii^—Sr2—O13^ix^	88.58 (11)	B1—O2—Sr4	97.3 (3)
O11^vi^—Sr2—O13^ii^	163.33 (10)	B7—O2—Sr4	129.7 (3)
O11^vii^—Sr2—O13^ii^	94.41 (10)	B1—O2—Sr4^xiv^	117.3 (3)
O11—Sr2—O13^ii^	75.13 (10)	B7—O2—Sr4^xiv^	91.4 (3)
O13^viii^—Sr2—O13^ii^	88.58 (11)	Sr4—O2—Sr4^xiv^	96.96 (10)
O13^ix^—Sr2—O13^ii^	88.58 (11)	B1—O3—B2	118.8 (4)
O11^vi^—Sr2—O15^viii^	48.77 (9)	B1—O3—Sr3	99.3 (3)
O11^vii^—Sr2—O15^viii^	77.18 (10)	B2—O3—Sr3	133.8 (3)
O11—Sr2—O15^viii^	148.54 (9)	B1—O3—Sr3^xiv^	134.9 (3)
O13^viii^—Sr2—O15^viii^	47.70 (9)	B2—O3—Sr3^xiv^	81.8 (3)
O13^ix^—Sr2—O15^viii^	89.33 (9)	Sr3—O3—Sr3^xiv^	89.54 (8)
O13^ii^—Sr2—O15^viii^	136.27 (10)	B2—O4—B7	122.2 (4)
O11^vi^—Sr2—O15^ix^	77.18 (10)	B2—O4—Sr5^xiv^	112.8 (3)
O11^vii^—Sr2—O15^ix^	148.54 (9)	B7—O4—Sr5^xiv^	117.1 (3)
O11—Sr2—O15^ix^	48.77 (9)	B2—O4—Sr5	112.8 (3)
O13^viii^—Sr2—O15^ix^	136.27 (10)	B7—O4—Sr5	89.7 (2)
O13^ix^—Sr2—O15^ix^	47.70 (9)	Sr5^xiv^—O4—Sr5	95.60 (10)
O13^ii^—Sr2—O15^ix^	89.33 (9)	B2—O5—B12^xv^	137.7 (4)
O15^viii^—Sr2—O15^ix^	119.955 (5)	B2—O5—Sr3^xiv^	112.1 (3)
O11^vi^—Sr2—O15^ii^	148.54 (9)	B12^xv^—O5—Sr3^xiv^	110.1 (2)
O11^vii^—Sr2—O15^ii^	48.77 (9)	B3—O6—B8	148.2 (4)
O11—Sr2—O15^ii^	77.18 (10)	B3—O6—Sr4	94.5 (3)
O13^viii^—Sr2—O15^ii^	89.33 (9)	B8—O6—Sr4	89.0 (2)
O13^ix^—Sr2—O15^ii^	136.27 (10)	B3—O6—Sr1	94.1 (3)
O13^ii^—Sr2—O15^ii^	47.70 (9)	B8—O6—Sr1	95.1 (2)
O15^viii^—Sr2—O15^ii^	119.955 (6)	Sr4—O6—Sr1	156.60 (14)
O15^ix^—Sr2—O15^ii^	119.955 (5)	B3—O7—B9	121.5 (4)
O5^x^—Sr3—O5^ii^	110.02 (8)	B3—O7—Sr4^xiv^	116.9 (3)
O5^x^—Sr3—O5^xi^	110.02 (8)	B9—O7—Sr4^xiv^	95.6 (2)
O5^ii^—Sr3—O5^xi^	110.02 (8)	B3—O7—Sr4	94.6 (3)
O5^x^—Sr3—O3^xii^	70.47 (10)	B9—O7—Sr4	127.1 (3)
O5^ii^—Sr3—O3^xii^	94.31 (9)	Sr4^xiv^—O7—Sr4	100.68 (10)
O5^xi^—Sr3—O3^xii^	152.90 (11)	B3—O8—B4	119.2 (4)
O5^x^—Sr3—O3	152.90 (11)	B3—O8—Sr1	100.4 (3)
O5^ii^—Sr3—O3	70.47 (10)	B4—O8—Sr1	132.7 (3)
O5^xi^—Sr3—O3	94.31 (9)	B3—O8—Sr1^xiv^	136.6 (3)
O3^xii^—Sr3—O3	82.43 (11)	B4—O8—Sr1^xiv^	79.8 (3)
O5^x^—Sr3—O3^xiii^	94.31 (9)	Sr1—O8—Sr1^xiv^	89.02 (8)
O5^ii^—Sr3—O3^xiii^	152.90 (11)	B4—O9—B9	123.2 (4)
O5^xi^—Sr3—O3^xiii^	70.47 (10)	B4—O9—Sr5^xvi^	114.8 (3)
O3^xii^—Sr3—O3^xiii^	82.43 (11)	B9—O9—Sr5^xvi^	120.0 (2)
O3—Sr3—O3^xiii^	82.43 (11)	B4—O10—B8^xvi^	135.4 (4)
O5^x^—Sr3—O1^xii^	77.41 (9)	B4—O10—Sr1^xiv^	114.0 (3)
O5^ii^—Sr3—O1^xii^	49.47 (9)	B8^xvi^—O10—Sr1^xiv^	110.6 (3)
O5^xi^—Sr3—O1^xii^	158.70 (11)	B5—O11—B10	131.2 (4)
O3^xii^—Sr3—O1^xii^	48.06 (9)	B5—O11—Sr2	114.5 (3)
O3—Sr3—O1^xii^	84.14 (10)	B10—O11—Sr2	112.6 (3)
O3^xiii^—Sr3—O1^xii^	129.98 (11)	B5—O12—B11	122.3 (4)
O5^x^—Sr3—O1^xiii^	49.47 (9)	B5—O12—Sr4	121.9 (3)
O5^ii^—Sr3—O1^xiii^	158.70 (10)	B11—O12—Sr4	115.1 (3)
O5^xi^—Sr3—O1^xiii^	77.41 (9)	B6—O13—B5	120.1 (4)
O3^xii^—Sr3—O1^xiii^	84.14 (10)	B6—O13—Sr2^xiv^	106.1 (3)
O3—Sr3—O1^xiii^	129.98 (11)	B5—O13—Sr2^xiv^	129.8 (3)
O3^xiii^—Sr3—O1^xiii^	48.06 (9)	B6—O14—B11	121.3 (4)
O1^xii^—Sr3—O1^xiii^	119.572 (18)	B6—O14—Sr5^xiii^	119.7 (3)
O5^x^—Sr3—O1	158.70 (11)	B11—O14—Sr5^xiii^	95.3 (3)
O5^ii^—Sr3—O1	77.41 (9)	B6—O14—Sr5^xvii^	89.9 (3)
O5^xi^—Sr3—O1	49.47 (9)	B11—O14—Sr5^xvii^	130.0 (3)
O3^xii^—Sr3—O1	129.98 (11)	Sr5^xiii^—O14—Sr5^xvii^	101.42 (10)
O3—Sr3—O1	48.06 (9)	B6—O15—B10^xviii^	147.5 (4)
O3^xiii^—Sr3—O1	84.14 (9)	B6—O15—Sr5^xvii^	98.7 (3)
O1^xii^—Sr3—O1	119.572 (19)	B10^xviii^—O15—Sr5^xvii^	93.8 (2)
O1^xiii^—Sr3—O1	119.572 (19)	B6—O15—Sr2^xiv^	87.5 (3)
O5^x^—Sr3—O3^x^	44.45 (9)	B10^xviii^—O15—Sr2^xiv^	89.5 (2)
O5^ii^—Sr3—O3^x^	68.82 (9)	Sr5^xvii^—O15—Sr2^xiv^	162.45 (13)
O5^xi^—Sr3—O3^x^	109.89 (10)	B8—O16—B7	125.3 (4)
O3^xii^—Sr3—O3^x^	89.54 (8)	B8—O16—Sr5	113.2 (3)
O3—Sr3—O3^x^	137.69 (4)	B7—O16—Sr5	111.1 (3)
O3^xiii^—Sr3—O3^x^	137.69 (4)	B8—O17—B7^ii^	137.0 (4)
O1^xii^—Sr3—O3^x^	60.73 (9)	B8—O17—Sr4	106.5 (3)
O1^xiii^—Sr3—O3^x^	89.91 (9)	B7^ii^—O17—Sr4	114.7 (3)
O1—Sr3—O3^x^	129.76 (10)	B9—O18—B10	133.6 (4)
O5^x^—Sr3—O3^xi^	68.82 (9)	B9—O18—Sr5^iv^	123.3 (3)
O5^ii^—Sr3—O3^xi^	109.89 (10)	B10—O18—Sr5^iv^	99.5 (3)
O5^xi^—Sr3—O3^xi^	44.45 (9)	B10—O19—B9^ii^	125.1 (4)
O3^xii^—Sr3—O3^xi^	137.69 (4)	B10—O19—Sr4	123.0 (3)
O3—Sr3—O3^xi^	137.69 (4)	B9^ii^—O19—Sr4	104.1 (3)
O3^xiii^—Sr3—O3^xi^	89.54 (8)	B12—O20—B11	130.6 (4)
O1^xii^—Sr3—O3^xi^	129.76 (10)	B12—O20—Sr5^xiii^	120.1 (3)
O1^xiii^—Sr3—O3^xi^	60.73 (9)	B11—O20—Sr5^xiii^	104.0 (3)
O1—Sr3—O3^xi^	89.91 (9)	B11^ii^—O21—B12	128.2 (4)
O3^x^—Sr3—O3^xi^	69.12 (9)	O1—B1—O2	121.8 (5)
O5^x^—Sr3—O3^ii^	109.89 (10)	O1—B1—O3	116.7 (4)
O5^ii^—Sr3—O3^ii^	44.45 (9)	O2—B1—O3	121.1 (4)
O5^xi^—Sr3—O3^ii^	68.82 (9)	O5—B2—O4	126.2 (4)
O3^xii^—Sr3—O3^ii^	137.69 (4)	O5—B2—O3	112.9 (4)
O3—Sr3—O3^ii^	89.54 (8)	O4—B2—O3	120.6 (4)
O3^xiii^—Sr3—O3^ii^	137.69 (4)	O6—B3—O7	122.4 (4)
O1^xii^—Sr3—O3^ii^	89.91 (9)	O6—B3—O8	116.6 (4)
O1^xiii^—Sr3—O3^ii^	129.76 (10)	O7—B3—O8	120.5 (4)
O1—Sr3—O3^ii^	60.73 (9)	O10—B4—O9	125.9 (4)
O3^x^—Sr3—O3^ii^	69.12 (9)	O10—B4—O8	113.1 (4)
O3^xi^—Sr3—O3^ii^	69.12 (9)	O9—B4—O8	120.7 (4)
O19—Sr4—O21	105.01 (10)	O11—B5—O12	126.8 (4)
O19—Sr4—O17	122.38 (10)	O11—B5—O13	112.6 (4)
O21—Sr4—O17	111.46 (10)	O12—B5—O13	120.4 (4)
O19—Sr4—O7^ii^	53.90 (9)	O15—B6—O14	120.4 (4)
O21—Sr4—O7^ii^	104.90 (10)	O15—B6—O13	118.6 (4)
O17—Sr4—O7^ii^	74.20 (10)	O14—B6—O13	120.1 (4)
O19—Sr4—O2	142.35 (11)	O17^xiv^—B7—O16	115.6 (4)
O21—Sr4—O2	100.85 (10)	O17^xiv^—B7—O2	104.0 (4)
O17—Sr4—O2	70.44 (10)	O16—B7—O2	110.3 (4)
O7^ii^—Sr4—O2	141.90 (9)	O17^xiv^—B7—O4	110.8 (4)
O19—Sr4—O12	77.05 (9)	O16—B7—O4	106.4 (4)
O21—Sr4—O12	74.24 (10)	O2—B7—O4	109.7 (4)
O17—Sr4—O12	154.69 (11)	O17—B8—O16	116.4 (4)
O7^ii^—Sr4—O12	129.48 (9)	O17—B8—O10^i^	111.8 (4)
O2—Sr4—O12	84.31 (10)	O16—B8—O10^i^	109.5 (4)
O19—Sr4—O1	149.31 (10)	O17—B8—O6	103.8 (4)
O21—Sr4—O1	50.97 (9)	O16—B8—O6	112.0 (4)
O17—Sr4—O1	87.17 (10)	O10^i^—B8—O6	102.2 (3)
O7^ii^—Sr4—O1	141.53 (10)	O18—B9—O19^xiv^	116.7 (4)
O2—Sr4—O1	50.18 (10)	O18—B9—O9	105.9 (4)
O12—Sr4—O1	77.65 (9)	O19^xiv^—B9—O9	110.2 (4)
O19—Sr4—O7	72.72 (9)	O18—B9—O7	110.4 (4)
O21—Sr4—O7	145.82 (10)	O19^xiv^—B9—O7	104.4 (3)
O17—Sr4—O7	96.98 (10)	O9—B9—O7	109.2 (4)
O7^ii^—Sr4—O7	100.68 (10)	O19—B10—O18	116.4 (4)
O2—Sr4—O7	70.49 (10)	O19—B10—O11	110.3 (4)
O12—Sr4—O7	72.05 (9)	O18—B10—O11	110.7 (4)
O1—Sr4—O7	115.02 (10)	O19—B10—O15^ix^	111.6 (4)
O19—Sr4—O6	93.03 (10)	O18—B10—O15^ix^	102.4 (3)
O21—Sr4—O6	159.89 (10)	O11—B10—O15^ix^	104.5 (3)
O17—Sr4—O6	49.69 (10)	O21^xiv^—B11—O20	116.5 (4)
O7^ii^—Sr4—O6	78.44 (10)	O21^xiv^—B11—O14	110.8 (4)
O2—Sr4—O6	68.12 (10)	O20—B11—O14	104.6 (4)
O12—Sr4—O6	119.38 (10)	O21^xiv^—B11—O12	106.0 (4)
O1—Sr4—O6	114.52 (9)	O20—B11—O12	109.2 (4)
O7—Sr4—O6	48.38 (9)	O14—B11—O12	109.7 (4)
O19—Sr4—O2^ii^	118.18 (10)	O20—B12—O21	116.5 (4)
O21—Sr4—O2^ii^	66.73 (10)	O20—B12—O1	111.7 (4)
O17—Sr4—O2^ii^	48.74 (9)	O21—B12—O1	104.4 (4)
O7^ii^—Sr4—O2^ii^	68.92 (9)	O20—B12—O5^xi^	109.0 (4)
O2—Sr4—O2^ii^	96.96 (10)	O21—B12—O5^xi^	111.8 (4)
O12—Sr4—O2^ii^	140.51 (9)	O1—B12—O5^xi^	102.5 (3)
O1—Sr4—O2^ii^	73.46 (9)		

Symmetry codes: (i) −*y*, *x*−*y*, *z*+1; (ii) *x*, *y*, *z*+1; (iii) −*x*+*y*, −*x*, *z*+1; (iv) −*x*+*y*, −*x*, *z*; (v) −*y*, *x*−*y*, *z*; (vi) −*y*+1, *x*−*y*, *z*; (vii) −*x*+*y*+1, −*x*+1, *z*; (viii) −*x*+*y*+1, −*x*+1, *z*+1; (ix) −*y*+1, *x*−*y*, *z*+1; (x) −*x*+*y*, −*x*+1, *z*+1; (xi) −*y*+1, *x*−*y*+1, *z*+1; (xii) −*x*+*y*, −*x*+1, *z*; (xiii) −*y*+1, *x*−*y*+1, *z*; (xiv) *x*, *y*, *z*−1; (xv) −*x*+*y*, −*x*+1, *z*−1; (xvi) −*x*+*y*, −*x*, *z*−1; (xvii) −*y*+1, *x*−*y*+1, *z*−1; (xviii) −*x*+*y*+1, −*x*+1, *z*−1.
